# Bipartite Community Structure of eQTLs

**DOI:** 10.1371/journal.pcbi.1005033

**Published:** 2016-09-12

**Authors:** John Platig, Peter J. Castaldi, Dawn DeMeo, John Quackenbush

**Affiliations:** 1 Department of Biostatistics and Computational Biology, Dana-Farber Cancer Institute, Boston, Massachusetts, United States of America; 2 Department of Biostatistics, Harvard Chan School of Public Health, Boston, Massachusetts, United States of America; 3 Channing Division of Network Medicine, Brigham and Women’s Hospital, Boston, Massachusetts, United States of America; 4 Division of General Medicine, Brigham and Women’s Hospital, Boston, Massachusetts, United States of America; 5 Harvard Medical School, Boston, Massachusetts, United States of America; 6 Division of Pulmonary and Critical Care Medicine, Brigham and Women’s Hospital, Boston, Massachusetts, United States of America; University of Cambridge, UNITED KINGDOM

## Abstract

Genome Wide Association Studies (GWAS) and expression quantitative trait locus (eQTL) analyses have identified genetic associations with a wide range of human phenotypes. However, many of these variants have weak effects and understanding their combined effect remains a challenge. One hypothesis is that multiple SNPs interact in complex networks to influence functional processes that ultimately lead to complex phenotypes, including disease states. Here we present CONDOR, a method that represents both *cis-* and *trans-*acting SNPs and the genes with which they are associated as a bipartite graph and then uses the modular structure of that graph to place SNPs into a functional context. In applying CONDOR to eQTLs in chronic obstructive pulmonary disease (COPD), we found the global network “hub” SNPs were devoid of disease associations through GWAS. However, the network was organized into 52 communities of SNPs and genes, many of which were enriched for genes in specific functional classes. We identified local hubs within each community (“core SNPs”) and these were enriched for GWAS SNPs for COPD and many other diseases. These results speak to our intuition: rather than single SNPs influencing single genes, we see groups of SNPs associated with the expression of families of functionally related genes and that disease SNPs are associated with the perturbation of those functions. These methods are not limited in their application to COPD and can be used in the analysis of a wide variety of disease processes and other phenotypic traits.

## Author Summary

Large-scale studies have identified thousands of genetic variants associated with different phenotypes without explaining their function. Expression quantitative trait locus analysis associates the compendium of genetic variants with expression levels of individual genes, providing the opportunity to link those variants to functions. But the complexity of those associations has caused most analyses to focus solely on genetic variants immediately adjacent to the genes they may influence. We describe a method that embraces the complexity, representing all variant-gene associations as a bipartite graph. The graph contains highly modular, functional communities in which disease-associated variants emerge as those likely to perturb the structure of the network and the function of the genes in these communities.

## Introduction

Genome Wide Association Studies (GWAS) have created new opportunities to understand the genetic factors that influence complex traits. Excepting highly-penetrant Mendelian disorders, the majority of genetic associations seem to be driven by many factors, each of which has a relatively small effect. In a recent study [1], 697 SNPs were associated with height in humans at genome-wide significance, yet these SNPs were able to explain only ∼20% of height variability; ∼9,500 SNPs were needed to raise that to ∼29%. In addition, ∼95% of GWAS variants map to non-coding regions [2], complicating biological interpretation of their functional impact.

To bridge the functional gap between genetic variant and complex trait, expression Quantitative Trait Locus (eQTL) analysis associates SNP genotype with gene expression levels. The first empirical, genome-wide linkage study with gene expression in yeast was published in 2002, linking expression levels of 570 genes to genetic loci [3]. In humans, loci have been associated with the expression of thousands of genes [2, 4], and eQTLs are enriched for phenotype associations and vice versa [5–7].

Most eQTL analyses have focused on *cis-*SNPs—those near the Transcriptional Start Site (TSS) of the gene in the association test. Recent computational developments [8] and work demonstrating the impact and replicability of *trans*-eQTLs [9, 10] have increased interest in identifying and understanding the role played by *trans-*acting SNPs.

**Fig 1:**
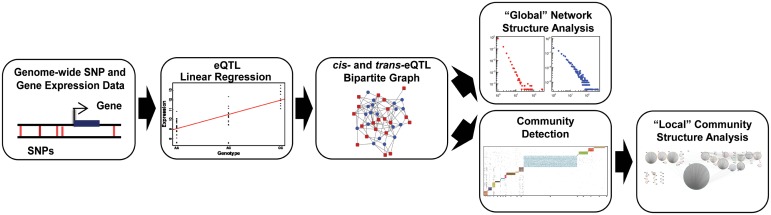
Overview of the CONDOR algorithm. All possible SNP-gene pairs from an appropriate data set are considered in an eQTL analysis. Both *cis-* and *trans-*acting eQTLs (FDR < 0.1) are used to construct a bipartite network linking SNPs and genes. The resulting network structure is then analyzed, first globally to understand its overall structure and to identify network “hubs.” Then the community structure of the bipartite network is determined, each community is subject to functional enrichment analysis, and a core score is calculated to identify those SNPs most likely to disrupt individual communities.

However, new methods are needed to elucidate the potential functional impact of the thousands of GWAS SNPs and tens to hundreds of thousands of eQTL SNPs that can be detected in a single study. Here we present CONDOR, COmplex Network Description Of Regulators, (Fig 1) a method that incorporates both *cis-* and *trans-* associations to identify groups of SNPs that are linked to groups of genes and systematically interrogate their biological functions. The method has been implemented as an R package and is publicly available at https://github.com/jplatig/condor. We then validate this approach using genotyping and gene expression data from 163 lung tissue samples in a study of Chronic Obstructive Pulmonary Disease (COPD) by the Lung Genomics Research Consortium (LGRC).

## Results

### eQTL Networks

**Fig 2:**
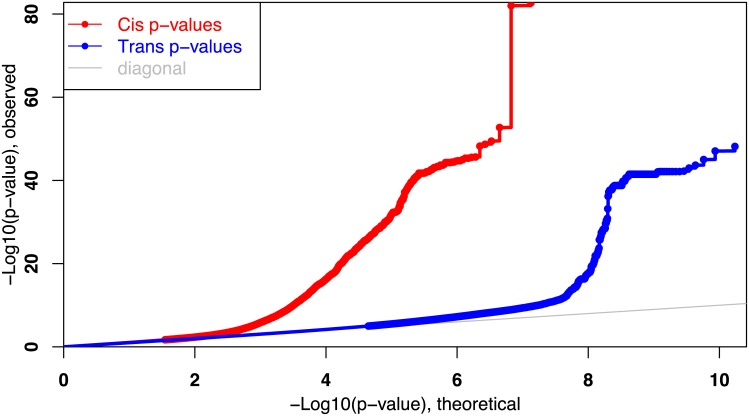
Quantile-quantile plot for 13,333,199 *cis-* and 17,228,062,483 *trans-*eQTL p-values.

We used the MatrixEQTL package in R to calculate *cis*- and *trans*-eQTLs, considering only autosomal SNPs, using age, sex, and pack-years as covariates (see Methods). The *cis-* and *trans-* associations were run separately, with an FDR threshold of 10%. This analysis identified 40,183 *cis*-eQTLs and 32,813 *trans*-eQTLs. Quantile-quantile plots for both *cis-* and *trans-* are shown in Fig 2. In total, 72,996 statistically significant associations were detected between 57,062 SNPs and 7,051 genes.

**Fig 3:**
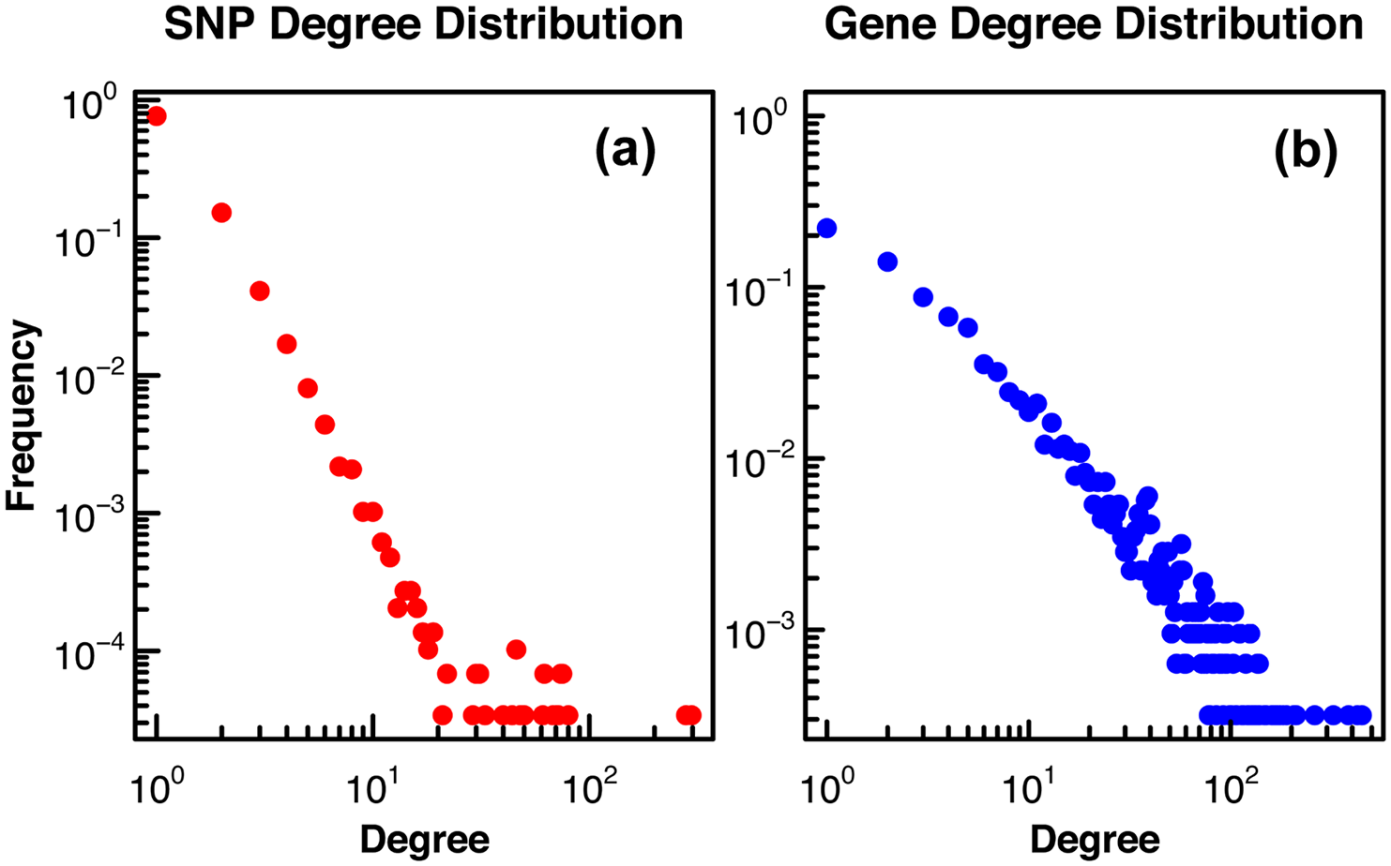
SNPs and genes display broad-tailed degree distributions. The degree distribution, with the frequency of node degree plotted on a log-log scale, is shown for SNPs **(a)** and genes **(b)** in all connected components with more than 5 SNPs and 5 genes in the bipartite eQTL network.

We represented these associations as a bipartite network consisting of two classes of nodes—SNPs and genes—with edges from SNPs to the genes with which they are significantly associated based on the eQTL FDR cut-off. The network had a Giant Connected Component (GCC) with 41,813 links, 28,593 SNPs, and 3,091 genes. As a network diagnostic, we estimated whether or not we could reject the hypothesis that the SNP and gene degree distributions were power-law distributed. To test this, we fit each degree distribution to a power law, and determined the goodness of fit using the method described in [11] (see Methods). If the edges from all connected components are considered, the p-value for the SNP degree is very low, *P*_*pl*_ ≈ 0, suggesting that we can rule out a power law distribution. However, if very small connected components (fewer than 5 SNPs and 5 genes) are excluded, the SNP degree may follow a power-law (*P*_*pl*_ < 0.8) as shown in Fig 3a. The gene degree distribution (Fig 3b) may be power-law distributed when considering all connected components or only those with more that 5 SNPs and 5 genes (*P*_*pl*_ < 0.4 in both cases) and there are multiple network hubs, shown in the tail of the distribution in Fig 3b. For our further analysis we considered all connected components with more than 5 SNPs and 5 genes.

**Fig 4:**
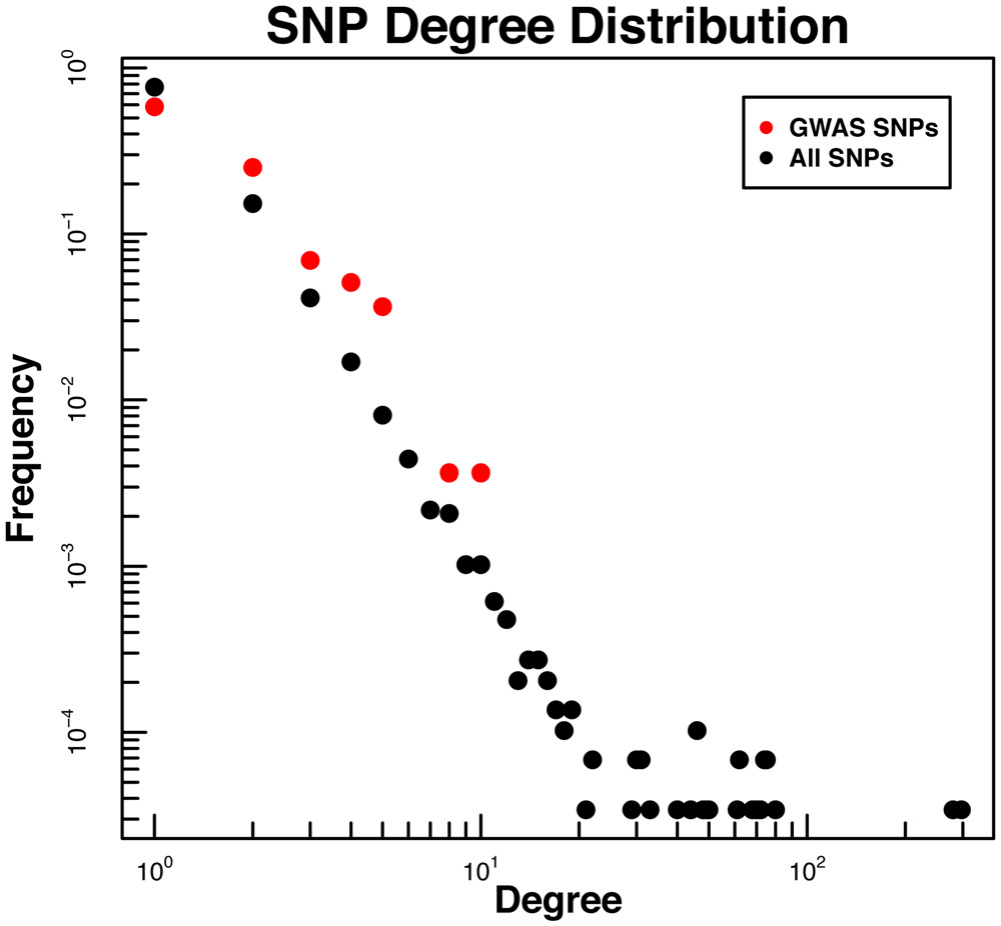
Degree distributions for NHGRI-GWAS (red) and all (black) SNPs. NHGRI-GWAS SNPs tend not to be global network “hubs,” which are located in the far-right tail of the distribution. The highest degree NHGRI-GWAS SNP was connected to 10 genes.

It is often cited in complex networks literature that the hubs, those nodes in the network that are most highly connected, represent critical elements whose removal can disrupt the entire network [12, 13]. As a result, one widely-held belief about biological networks is that disease-related elements should be over-represented among the network hubs [14]. To test the hypothesis that disease-associated SNPs are concentrated in the hubs, we projected GWAS-identified SNPs associated with a wide range of diseases and phenotypes onto the SNP degree distribution (Fig 4). We used the *gwascat* package [15] in R to download GWAS SNPs annotated in the NHGRI GWAS catalog; 274 of those SNPs mapped to the eQTL network (S1 Table). To our surprise, the network hubs—the right tail of Fig 4—were devoid of disease-associated SNPs which were instead scattered through the upper left half of the degree distribution. The difference in degree distributions did not appear to be driven by linkage disequilibrium or distance to nearest gene (see Methods and S1, S2, S3 and S4 Figs). While the SNPs associated with a single gene are easier to interpret, the concentration of disease-associated SNPs in the middle of the distribution prompted us to look at other features of the network and its structure.

### Community Structure Analysis

Given the low phenotypic variance explained by any single GWAS SNP, we expected groups of SNPs to cluster with groups of functionally-related genes in our eQTL network. Unlike previous work [16–18] which imposes “known” pathway annotations and other data to posit the function of GWAS SNPs or identifies modules with only a handful of SNPs [19], we used the structure of the eQTL network to identify densely connected groups of SNPs and genes and then tested those groups for biological enrichment.

Our goal is the identification of those densely connected communities in the bipartite network. Methods for finding bicliques (subgraphs with all-to-all connections within the larger bipartite network) have been described for bipartite networks with a small number (∼10^2^) of nodes in each connected component [20]. However, these methods do not scale to networks with connected components containing thousands of nodes [20, 21]. Further, we do not expect biologically meaningful eQTL clusters to contain only all-to-all connections.

To cluster our eQTL network, we adapted a well-established strategy [22], community structure detection, which has been shown to scale well to large networks [23]. Many real-world networks have a complex structure consisting of “communities” of nodes [24]. These communities are often defined as a group of network nodes that are more likely to be connected to other nodes within their community than they are to those outside of the community. A widely used measure of community structure is the modularity, which can be interpreted as an enrichment for links within communities minus an expected enrichment given the network degree distribution [22].

To partition the nodes from the eQTL network into communities—which contain both SNPs and genes—we maximized the bipartite modularity [25]. As recursive cluster identification and optimization can be computationally slow, we calculated an initial community structure assignment on the weighted, gene-space projection, using a fast uni-partite modularity maximization algorithm [23] available in the R *igraph* package [26], then iteratively converged (Δ*Q* < 10^−4^) on a community structure corresponding to a maximum bipartite modularity.

The bipartite modularity is defined in Eq (1), where *m* is the number of links in the network, ${\tilde{A}}_{ij}$ is the upper right block of the network adjacency matrix (a binary matrix where a 1 represents a connection between a SNP and a gene and 0 otherwise), *k*_*i*_ is the degree of SNP *i*, *d*_*j*_ is the degree of gene *j*, and *C*_*i*_, *C*_*j*_ the community indices of SNP *i* and gene *j*, respectively (see [25] for further details).

$$Q=\frac{1}{m}\sum_{i,j}\left({\tilde{A}}_{ij}-\frac{k_{i}d_{j}}{m}\right)\delta \left(C_{i},C_{j}\right)$$ (1)

**Fig 5:**
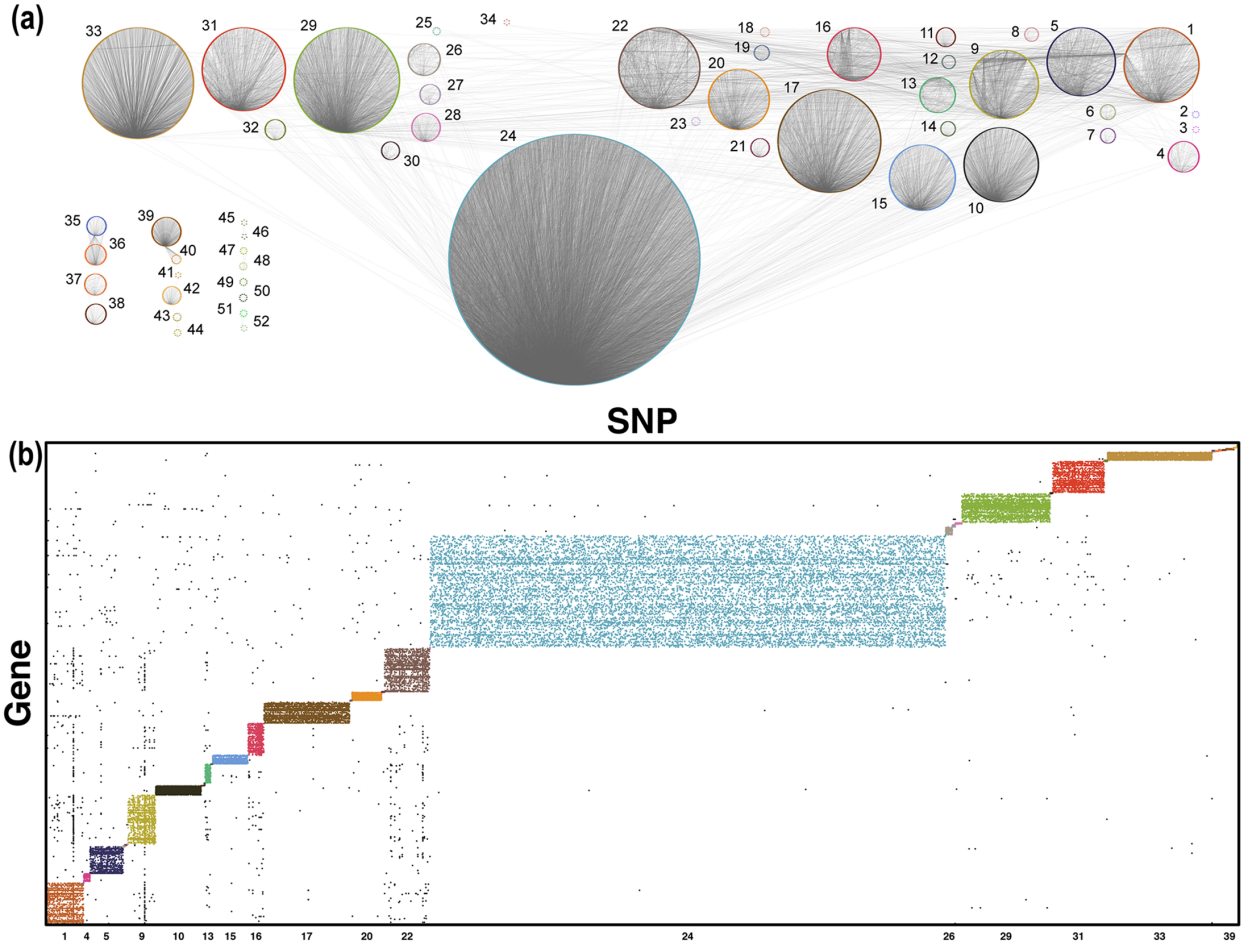
eQTLs show strong community structure. **(a)** Plot of the communities within the bipartite eQTL network. The nodes (genes and SNPs) in each community form a ring, with the link density within each ring visibly darker than links between communities. **(b)** Links within communities (colored points) are shown along the diagonal, with links that go between communities in black. Community IDs are plotted along the *x*-axis.

**Fig 6:**
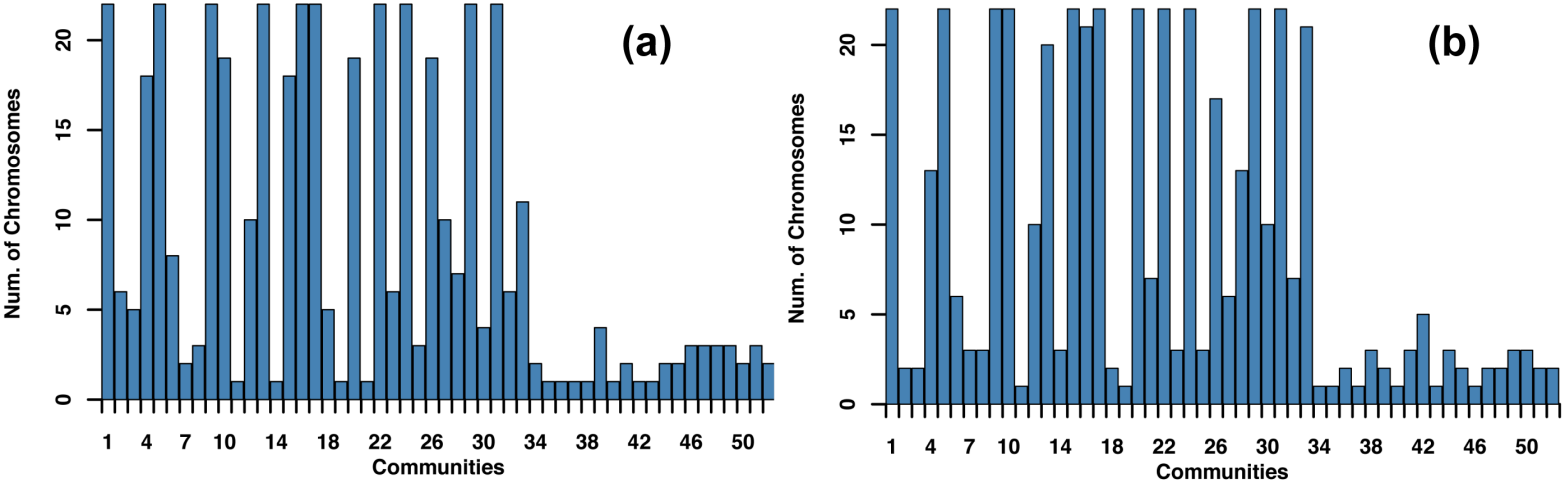
Communities comprise SNPs and genes from multiple chromosomes. Number of different chromosomes in each community based on (a) SNP and (b) gene locations.

This analysis identified 52 communities across 10 connected components in the LGRC data, with 34 of those communities mapping to the GCC (*Q*_*gcc*_ = 0.79; Fig 5). The density of these communities can be seen in Fig 5. In Fig 5b, there is visible enrichment for links within each community (colored links) compared to links between different communities (black links). These communities represent groups of SNPs and genes that are highly connected to each other and span multiple chromosomes (see Fig 6), suggesting that groups of genes may be jointly moderated by groups of SNPs that together represent specific biological processes.

To investigate this hypothesis, we tested each community for GO term enrichment using Fisher’s Exact Test (available in the R package *GOstats* [27]) and found 11 of the 52 communities contained genes enriched for specific Gene Ontology terms (see S2 Table) (*P* < 5*e* − 4; overlap >4), encompassing a broad collection of cellular functions that are not generally associated with COPD. Indeed, this is what one might expect as the genetic background of an individual should have an effect not only on disease-specific processes, but more globally on the physiology of his or her individual cells. A number of communities do, however, show enrichment for biological processes that are known to be involved in COPD, including genes previously associated with the disease.

For example, Community 29 (see Fig 5 and S2 Table) was enriched for chromatin and nucleosome assembly/organization and includes members of the HIST1H gene superfamily. Community 33 (see Fig 5 and S2 Table) included GO term enrichment for functions related to the HLA gene family, including T cell function and immune response; autoimmunity has been suggested as a potential contributor to COPD pathogenesis [28]. This community also contains *PSORS1C1*, which has been previously implicated in COPD [29].

Another of the genes in Community 33, *AGER*, has been implicated in COPD [30] and encodes sRAGE, a biomarker for emphysema. Its expression is negatively associated via eQTL analysis (*β* = −0.3) with rs6924102. This SNP has been observed to be an eQTL in a large blood eQTL dataset for a number of neighboring genes [9], but it has not previously been described as an eQTL for *AGER*. This SNP lies in a region containing a DNase peak in cell lines analyzed by ENCODE [31] (indicating it sits in a region of open chromatin) and there is evidence of POLR2A binding from ChIP-Seq data in the GM12878 cell line as reported by ENCODE (http://regulomedb.org/snp/chr6/32811382). This suggests that rs6924102 may inhibit the expression of *AGER* through disruption of RNA Polymerase II binding and subsequent mRNA synthesis. This SNP is located ∼700KB from the well-studied non-synonymous *AGER* SNP, rs2070600.

### Core Score Analysis

Examining Fig 5a, it is evident that within each community there are local hubs that are highly connected to the genes within that community. While a wide array of network node metrics exist (for example, [32, 33] and references in [33]), most of these metrics are global measures that do not consider a node’s role in its local cluster/community and so may miss SNPs that are central to their communities and therefore likely to alter gene expression of functionally associated genes. Such within-community hubs have been observed in protein-protein interaction networks [34] and metabolic networks [35].

We defined a core score that estimates importance of a SNP in the structure of its community. For SNP *i* in community *h*, its core score, *Q*_*ih*_, Eq (2), is the fraction of the modularity of community *h*, *Q*_*h*_, Eq (3), contributed by SNP *i*. This allows for comparison of SNPs from different communities, as each community does not have the same modularity, *Q*_*h*_.

$$Q_{ih}=\frac{\frac{1}{m}\sum_{j}\left({\tilde{A}}_{ij}-\frac{k_{i}d_{j}}{m}\right)\delta \left(C_{i},h\right)\delta \left(C_{j},h\right)}{Q_{h}}$$ (2)

$$Q_{h}=\frac{1}{m}\underset{i,j}{\sum }\left({\tilde{A}}_{ij}-\frac{k_{i}d_{j}}{m}\right)\delta \left(C_{i},h\right)\delta \left(C_{j},h\right)$$ (3)

If one views disease as the disruption of a process leading to cellular or organismal dysfunction, one natural hypothesis is that SNPs with the greatest potential to disrupt cellular processes might be enriched for disease association. To test this we used both the Wilcoxon rank-sum and Kolmogorov-Smirnov (KS) tests to assay whether the 274 NHGRI GWAS-annotated SNPs in the network were more likely to have high *Q*_*ih*_ scores. For both tests, the distribution of *Q*_*ih*_ scores for GWAS-associated SNPs were compared to the distribution of non-GWAS SNP scores.

**Fig 7:**
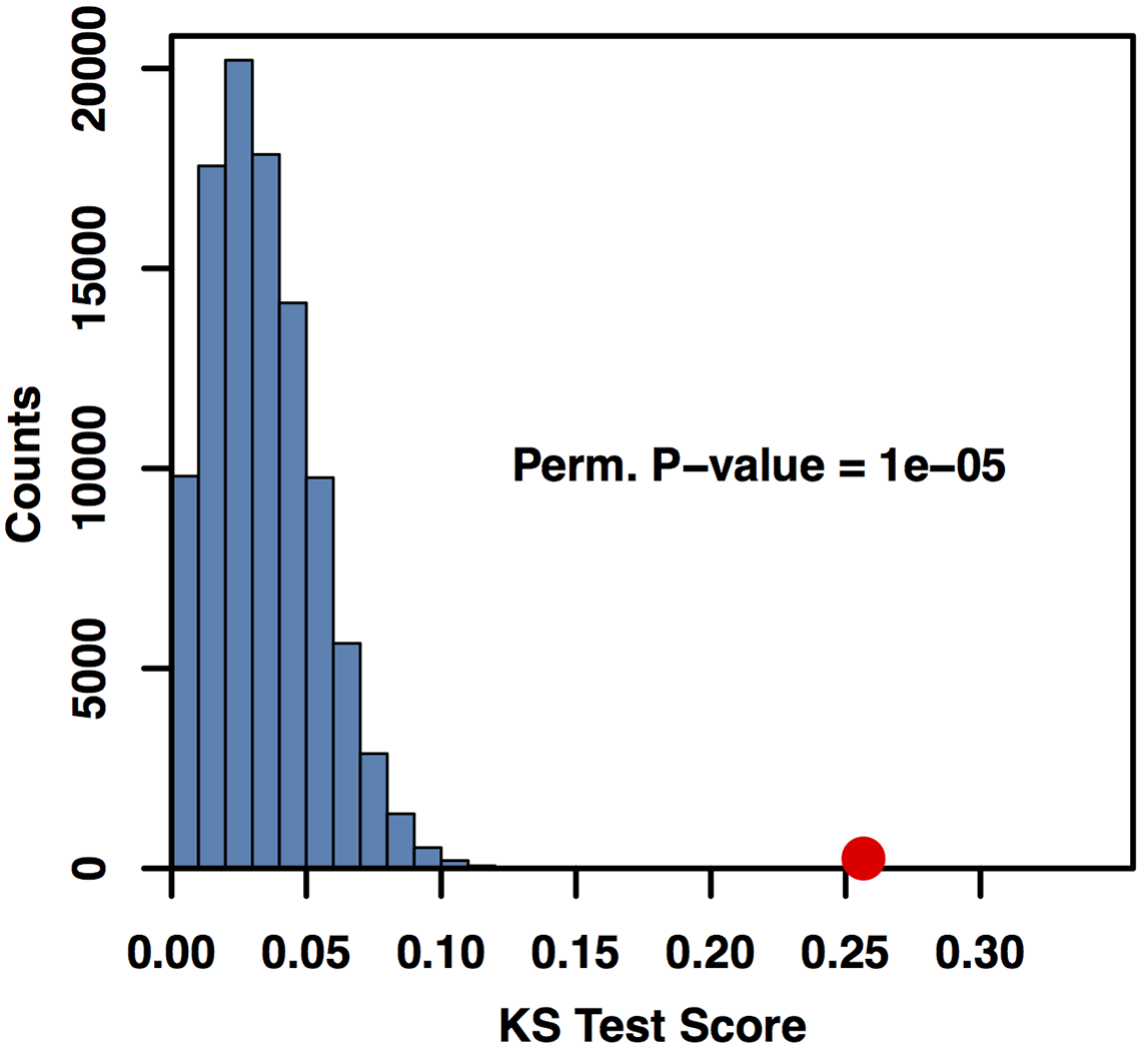
NHGRI-GWAS SNPs have higher core scores than non-GWAS SNPs based on Kolmogorov-Smirnov test statistics. Histogram of Kolmogorov-Smirnov test statistics comparing the distribution of *Q*_*ih*_ scores for sets of randomly relabeled NHGRI-GWAS/non-GWAS SNPs. The KS test statistic for the true labeling is in red. The permutation p-value associated with the KS test is *P* < 10^−5^ given 10^5^ permutations.

**Fig 8:**
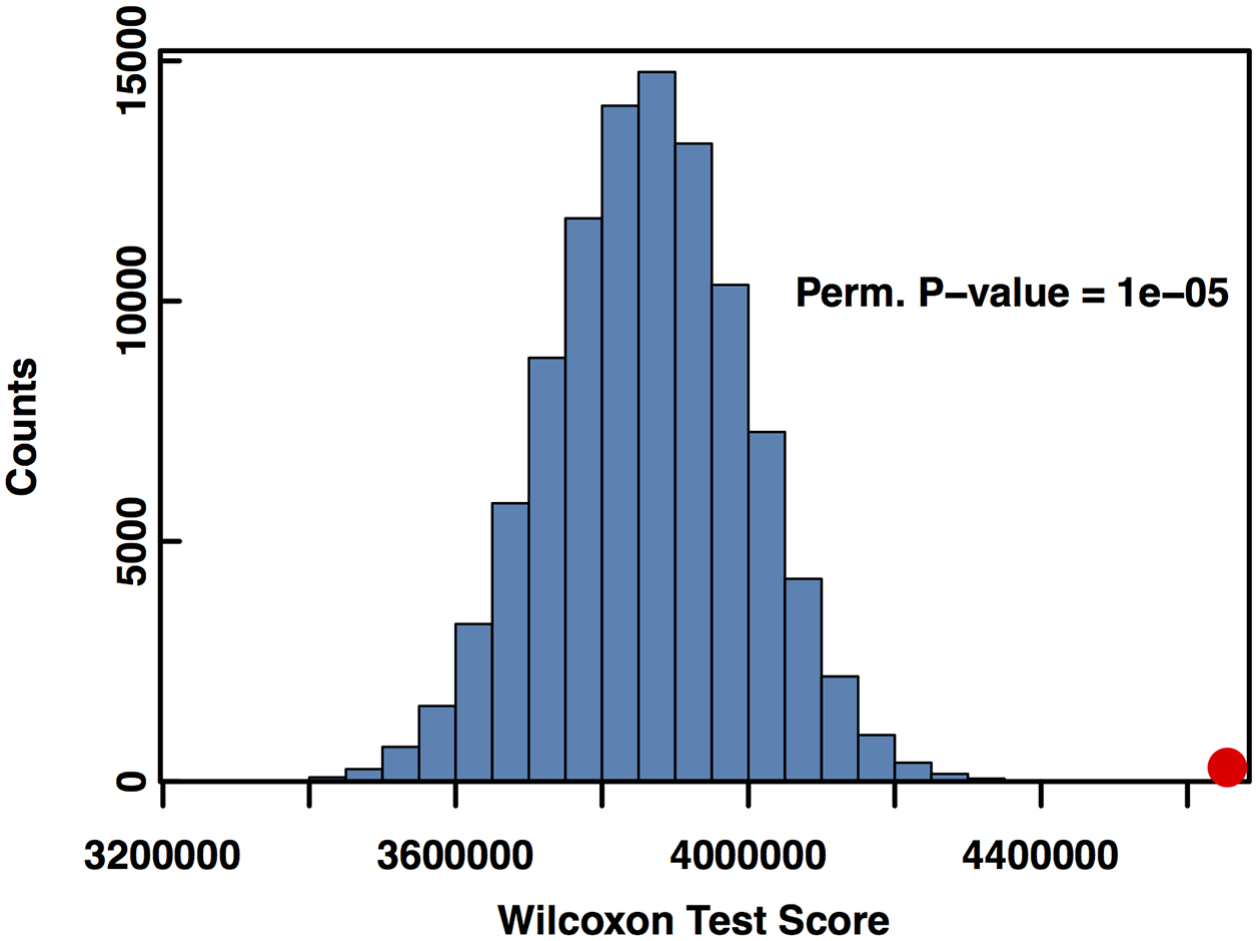
NHGRI-GWAS SNPs have higher core scores than non-GWAS SNPs based on Wilcoxon test statistics. Histogram of Wilcoxon test statistics comparing the distribution of *Q*_*ih*_ scores for sets of randomly relabeled NHGRI-GWAS/non-GWAS SNPs. The Wilcoxon test statistic for the true labeling is in red. The permutation p-value associated with the Wilcoxon test is *P* < 10^−5^ given 10^5^ permutations.

To obtain an empirical p-value for these tests, we permuted the GWAS/non-GWAS labels and recalculated the KS and Wilcoxon tests 10^5^ times. Histograms of the test statistics are shown in Figs 7 and 8. The red dot in the histogram represents the test score with the true labeling. Both tests had highly significant permutation p-values, with *P* < 10^−5^ for the KS and Wilcoxon tests, indicating that GWAS SNPs were over-represented among SNPs with high core scores. Furthermore, the median core score for the GWAS SNPs was 1.74 times higher than the median core score for the non-GWAS SNPs. To test this result for dependence on Linkage Disequilibrium (LD) and gene distance, we reran the KS and Wilcoxon permutation tests with a subset of SNPs matching the LD structure and distance to nearest gene of the 274 GWAS SNPs (see Methods for details). Neither the LD structure (*P* < 0.001 for KS and Wilcoxon tests, S5 and S6 Figs) nor distance from the nearest gene (*P* < 0.001 for KS and Wilcoxon tests, S7 and S8 Figs) of the GWAS SNPs was signficantly associated with the core score. Thus, while global hubs are devoid of GWAS associations with disease, local hubs within communities are significantly enriched for disease associations.

**Fig 9:**
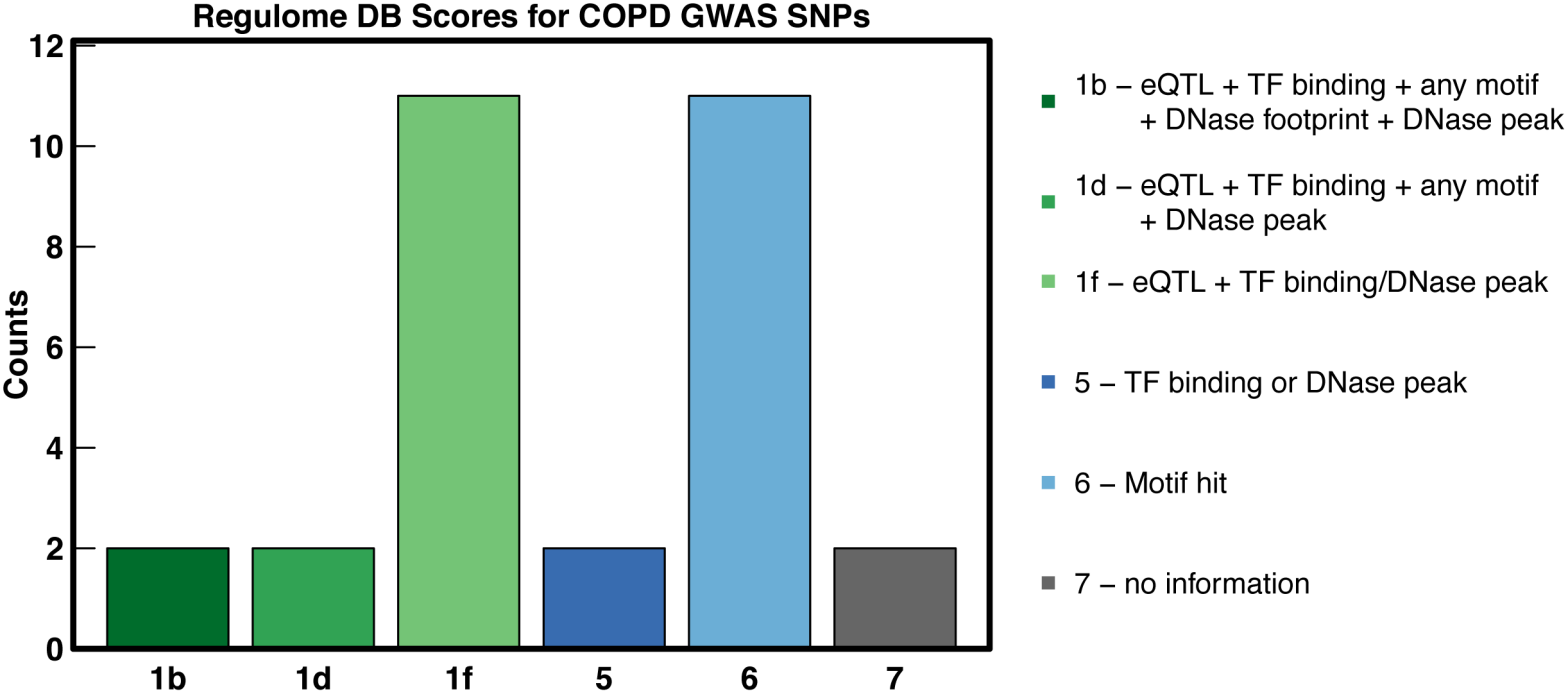
The majority of COPD Network GWAS SNPs are annotated for functional impact. Of the 30 SNPs that are eQTLs in the LGRC network and also associated with COPD (FDR < 0.05), 15 are likely to affect transcription factor (TF) binding and linked to the expression of a target gene (a score of 1b, d, or f), 2 have evidence of TF binding or a DNase peak (a score of 5), and 11 are located in a motif hit (a score of 6) according to RegulomeDB [37].

As a way of further assessing the link between GWAS significance and functional perturbation in COPD, we calculated a GWAS-FDR for all SNPs clustered in our network that had a reported p-value from a recent GWAS and meta-analysis of COPD [36] (see Methods). There were 30 SNPs with an FDR < 0.05, and 28 of the 30 had evidence of functional impact according to RegulomeDB [37], with 15 SNPs identified as likely to affect transcription factor binding and linked to expression (See Fig 9 and S3 Table). These 30 SNPs mapped to 3 different communities (see S3 Table) including Community 33, which contains other COPD-associated SNPs and genes, and is enriched for GO terms describing T cell function and immune response. One of the SNPs in this community likely to affect binding (S3 Table) is rs9268528, which is linked by our network to *HLA-DRA*, *HLA-DRB4*, and *HLA-DRB5*; the *cis-*eQTL associations between rs9268528 and both *HLA-DRA* and *HLA-DRB5* have been previously observed in lymphoblastoid cells [38]. All three HLA genes lie in Community 33 and contribute to the community’s enrichment for T cell receptor signaling pathway (GO:0050852) [39].

**Fig 10:**
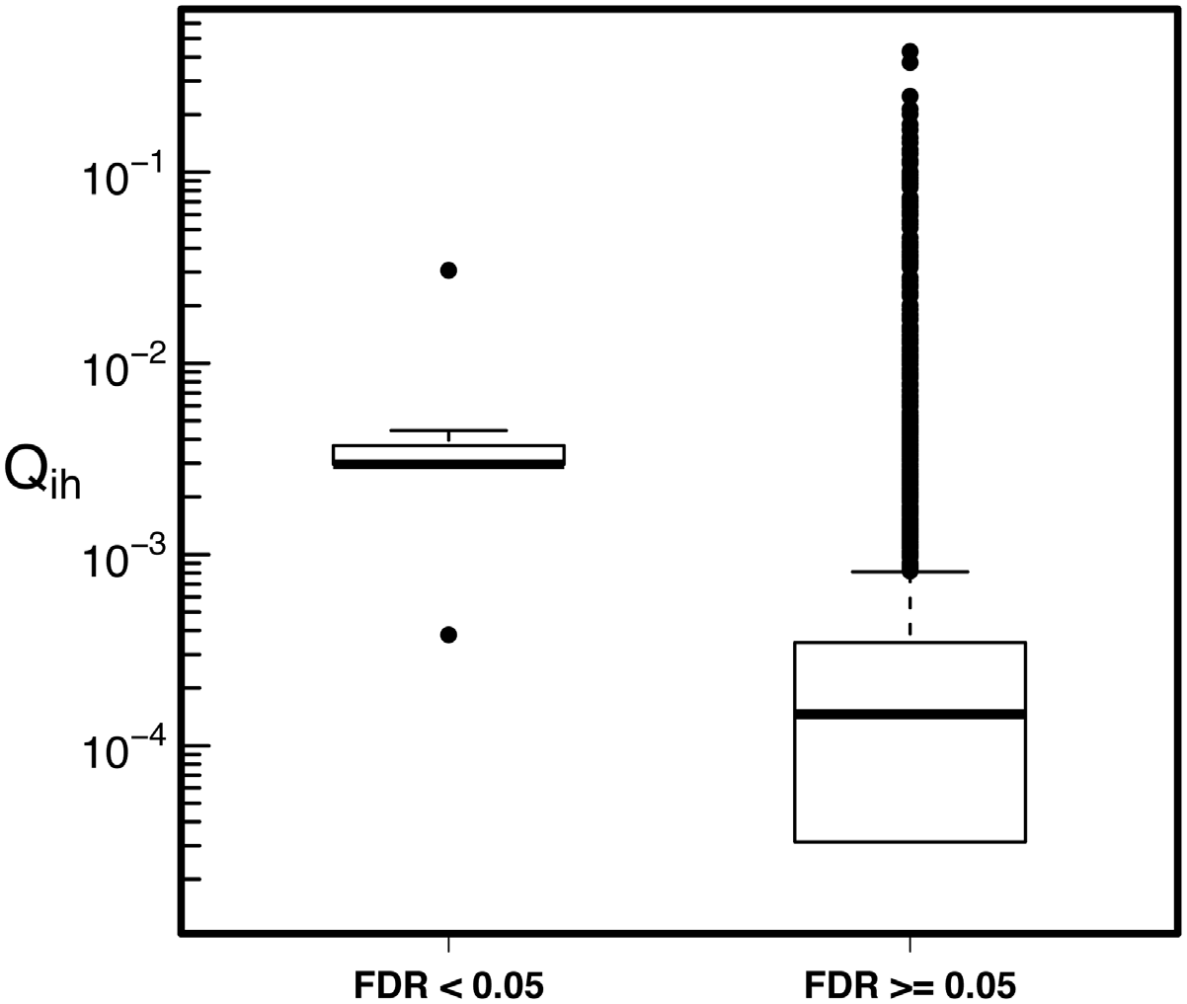
The median core score for COPD Network GWAS SNPs is higher than for non-significant SNPs. The median core score for the 30 FDR-significant COPD GWAS SNPs (FDR < 0.05, left) is 20.3 times higher than the median core score for the non-significant SNPs (FDR ≥ 0.05, right).

To determine the network influence of these 30 SNPs, we compared their core score, *Q*_*ih*_, to the core scores of SNPs with a GWAS-FDR ≥ 0.05 (See Fig 10). The median *Q*_*ih*_ value for the 30 GWAS-FDR significant SNPs was 20.3 times higher than the median for SNPs with an FDR ≥ 0.05. Using the KS and Wilcoxon tests described in the Methods, these core scores were not significantly associated with LD structure (*P* < 0.001, S9 and S10 Figs) or distance to nearest GSS (*P* < 0.001, S11 and S12 Figs).

### Conclusions

Genome-wide association studies have searched for genomic variants that influence complex traits, including the development and progression of disease. However, the number of highly-penetrant Mendelian variants that have been found is surprisingly small, with most disease-associated SNPs having a weak phenotypic effect. GWAS studies have also identified many SNPs that do not alter protein coding and have found significant loci that are shared in common across multiple diseases. This body of evidence suggests that in most instances it is not a single genetic variant that leads to disease, but many variants of smaller effect that together can disrupt cellular processes that lead to disease phenotypes. The challenge has been to find these variants of small effect and to place them into a coherent biological context.

We chose to address this problem by analyzing the link between genetic variants and the most immediate phenotypic measure, gene expression. In doing so, we chose not to focus solely on *cis-*acting SNPs, but also to consider *trans-*acting variants. Our motivation was, in part, to try to understand SNPs found through GWAS studies to be associated with phenotypes, but that could not be immediately placed into a functional context. After performing a genome-wide *cis-* and *trans-*eQTL analysis, we identified a large number of many-to-many associations: single SNPs associated with many genes as well as single genes that were significantly associated with many SNPs. To represent those associations, we constructed a bipartite network, one that contains two types of nodes—SNPs and genes—with edges connecting SNPs to the genes with which they were significantly associated. Our analysis of that network led to a number of observations that independently speak to our intuition about disease and the genetic factors that control it.

First is the observation that the highly connected SNPs, the global hubs in the network, are devoid of variants that have been identified as being disease-associated in the hundreds of studies collected in the NHGRI GWAS catalog. While initially surprising, further consideration suggests that this may be the result of negative selection. Since a true hub SNP influences genes across the genome that are involved in many biological processes, highly disruptive variants that are hubs are likely to significantly affect cellular function. In fact, this is the expected impact of a hub—its disruption should lead to the catastrophic collapse of the network. And so, disruptive SNPs that would be network hubs are likely to be lethal or highly debilitating and therefore strongly selected against and quickly swept from the genome.

Second, we found that SNPs and their target genes form highly connected communities that are enriched for specific biological functions. This too speaks to our inituition and to the evidence about polygenic traits that has accumulated over time. They are not the result of a single SNP that regulates a single gene, but a family of SNPs that together help mediate a group of functionally-related genes.

Third, the enrichment for GWAS disease associations among the high core score SNPs has a very simple and intuitive interpretation. The SNPs that are most significantly connected within a particular functionally-related group are those most likely to disrupt that process and therefore be discovered in GWAS analysis. After all, diseases do not develop because the cell’s entire functionality collapses, but because specific processes within the cell are disrupted.

What our analysis provides is a new way of exploring *cis-* and *trans-*eQTL analysis and GWAS. What one must do is to consider not only the local effects of genetic variants, but also the complex network of genetic interactions that help regulate phenotypes, including gene expression.

### Future Directions

This method also suggests a new way of filtering genes for inclusion in GWAS analysis. Since many disease-associated SNPs appear to be either *cis-*acting or those which are central to functionally-defined communities, one could focus on those SNPs most likely perturb specific biological processes rather than considering the entirety of SNPs in the genome.

One might note that this analysis was carried out using data on genetic variation and gene expression from the LGRC representing COPD and control lung tissue and question both the generalizability of the results and the use of GWAS-associated disease SNPs from many diseases in the analysis. While these are potentially legitimate concerns, many of the community-based processes we find are not specific to COPD or to the lung but instead are active in nearly all human cell types.

Although one might expect some processes to change in different disease states, the impact of common variants and the structure of the network is likely to be highly similar. Consequently, although there may be some SNPs whose impact is disease- and tissue-specific, many are likely to be independent of disease state. This suggests that it may be useful to develop eQTL networks across disease states and tissue types and to explore changes in the overall network and community structure across and between phenotypes due to rare variants and tissue-specific expression.

Validating individual associations in the eQTL network is a difficult challenge. Most eQTL studies limit their validation efforts to downstream effects of high-confidence *cis-*acting eQTLs. The bipartite network presented here captures not only these strong *cis-*eQTLs but also the weak effects of many more *cis-* and *trans-*acting SNPs. So the likelihood that any individual association can be easily validated may not be that great, as it is likely to be of small phenotypic effect and important in only a subset of individuals. However, this is not the point. What is important for the phenotype is not any single SNP-gene association, but the “mesoscale” organization of genes and SNPs represented by the communities in the network. We believe this intermediate structure better reflects the aggregation of weak genetic effects that contribute to late-onset complex diseases. What we hope to have demonstrated in this manuscript is that the higher order structure, which was not an input to the network model, provides insight into a number of aspects of the genetics and manifestation of polygenic traits.

## Methods

We began by downloading gene expression data from the LGRC web portal (https://www.lung-genomics.org/download/) representing data from COPD-case and control samples generated by the Lung Genomics Research Consortium (LGRC). This included GCRMA-normalized gene expression data obtained using Agilent-014850 Whole Human Genome 4x44K and Agilent-028004 SurePrint G3 Human GE 8x60K Microarrays. We then obtained matching genotyping data (dbGAP accession phs000624.v1.p1) collected using the Illumina Infinium HD Assays with Human Omni 1 Quad and Human Omni 2.5 Quad arrays. All subjects were reported to be of Caucasian descent and were selected based on a variety of parameters including clinical measures associated with diagnosis. Samples that did not meet standards for lack of relatedness as measured using Identity by Descent (IBD) and inbreeding coefficient, *F*, were excluded. Those samples with discordance between reported and genetic sex were not included. Samples missing more than 10% percent of genotyped SNPs were also removed. SNPs with minor allele frequency (MAF) < 0.05 or Hardy Weinberg Equilibrium P-value < 0.001 were removed. After all quality controls, 163 samples remained. All SNPs were mapped to human genome 19, and the Ensembl IDs provided by the LGRC web portal were mapped to the GRCh37 build of human genome 19 using the biomaRt library [40] in R. The *cis-*window was defined as +/- 1 MB of the Ensembl-defined GSS. The COPD GWAS data from a meta-analysis of COPDGene non-Hispanic whites and African-Americans, ECLIPSE, GenKOLS, and NETT/NAS studies was obtained from the authors of [36]. The bipartite clustering via modularity maximization took 95 seconds on a 64-bit Linux server with 189 GB of RAM running R 3.1.3.

### Power-Law Fitting

For each empirical degree distribution, we fit the two parameters for a power-law: the minimum degree at which the power-law behavior starts, *d*_*min*_, and the exponent, *α*. A Kolmogorov-Smirnov test was then used to estimate the goodness of fit between 5,000 randomly generated power-law distributed synthetic data sets given *d*_*min*_ and *α* and their corresponding power-law fit. The p-value, *P*_*pl*_, used to reject the power-law hypothesis is then the fraction of times a synthetic data set has a KS statistic larger than that of the true test. For both the SNP and gene degree distributions, *P*_*pl*_ was calculated using the 5,000 goodness of fit values (code for the parameter estimation, goodness of fit and probability estimation was obtained from the website associated with [11]).

### Permutation Testing for LD and Gene Distance

To test the effect of LD and distance from Gene Start Site (GSS) on the degree distribution and core score (*Q*_*ih*_) distribution of a set of GWAS SNPs, we created equivalently sized sets of SNPs that matched on a given characteristic of interest (LD or GSS) and compared that subset to all other SNPs. We repeated this process for each GWAS SNP set 1000 times. For the LD testing, we calculated LD blocks using the PLINK [41] “blocks” flag, estimating blocks using all SNPs that passed quality control. To achieve adequate sample sizes in the resampling, we binned LD blocks in 5kb windows, grouped all blocks >100kb into one bin and grouped all SNPs not in a block into one bin. For each resampling, the random set matched the GWAS set for both the LD bin and the number of SNPs in LD together within a block.

As a proxy for the gene density of a region, we used each SNP’s distance from the nearest GSS. Distances were grouped into 1kb bins, with all distances >400kb grouped into one bin. The resampled sets were then matched on the GWAS SNP sets such that the number of SNPs in each bin was the same.

## Supporting Information

S1 Fig NHGRI-GWAS degree does not depend on LD structure using a KS test. Histogram of KS test statistics comparing the distribution of degrees for sets of SNPs matched on LD structure of the NHGRI-GWAS SNPs and all other SNPs. The test statistic for the true labeling is in red. The permutation p-value is *P* < 10^−3^ given 10^3^ permutations.
(EPS)

S2 Fig NHGRI-GWAS degree does not depend on LD structure using a Wilcoxon test. Histogram of Wilcoxon test statistics comparing the distribution of degrees for sets of SNPs matched on LD structure of the NHGRI-GWAS SNPs and all other SNPs. The test statistic for the true labeling is in red. The permutation p-value is *P* < 10^−3^ given 10^3^ permutations.
(EPS)

S3 Fig NHGRI-GWAS degree does not depend on distance to nearest gene using KS test. Histogram of KS test statistics comparing the distribution of degrees for sets of SNPs matched on distance to nearest gene start site (GSS) of the NHGRI-GWAS SNPs and all other SNPs. The test statistic for the true labeling is in red. The permutation p-value is *P* < 10^−3^ given 10^3^ permutations.
(EPS)

S4 Fig NHGRI-GWAS degree does not depend on distance to nearest gene using a Wilcoxon test. Histogram of Wilcoxon test statistics comparing the distribution of degrees for sets of SNPs matched on distance to nearest gene start site (GSS) of the NHGRI-GWAS SNPs and all other SNPs. The test statistic for the true labeling is in red. The permutation p-value is *P* < 10^−3^ given 10^3^ permutations.
(EPS)

S5 Fig NHGRI-GWAS *Q*_*ih*_ scores do not depend on LD structure using a KS test. Histogram of KS test statistics comparing the distribution of *Q*_*ih*_ scores for sets of SNPs matched on LD structure of the NHGRI-GWAS SNPs and all other SNPs. The test statistic for the true labeling is in red. The permutation p-value is *P* < 10^−3^ given 10^3^ permutations.
(EPS)

S6 Fig NHGRI-GWAS *Q*_*ih*_ scores do not depend on LD structure using a Wilcoxon test. Histogram of Wilcoxon test statistics comparing the distribution of *Q*_*ih*_ scores for sets of SNPs matched on LD structure of the NHGRI-GWAS SNPs and all other SNPs. The test statistic for the true labeling is in red. The permutation p-value is *P* < 10^−3^ given 10^3^ permutations.
(EPS)

S7 Fig NHGRI-GWAS *Q*_*ih*_ scores do not depend on distance to nearest gene using a KS test. Histogram of KS test statistics comparing the distribution of *Q*_*ih*_ scores for sets of SNPs matched on distance to nearest GSS of the NHGRI-GWAS SNPs and all other SNPs. The test statistic for the true labeling is in red. The permutation p-value is *P* < 10^−3^ given 10^3^ permutations.
(EPS)

S8 Fig NHGRI-GWAS *Q*_*ih*_ scores do not depend on distance to nearest gene using a Wilcoxon test. Histogram of Wilcoxon test statistics comparing the distribution of *Q*_*ih*_ scores for sets of SNPs matched on distance to nearest GSS of the NHGRI-GWAS SNPs and all other SNPs. The test statistic for the true labeling is in red. The permutation p-value is *P* < 10^−3^ given 10^3^ permutations.
(EPS)

S9 Fig COPD GWAS *Q*_*ih*_ scores do not depend on LD structure using a KS test. Histogram of KS test statistics comparing the distribution of *Q*_*ih*_ scores for sets of SNPs matched on LD structure of the COPD GWAS SNPs and all other SNPs. The test statistic for the true labeling is in red. The permutation p-value is *P* < 10^−3^ given 10^3^ permutations.
(EPS)

S10 Fig COPD GWAS *Q*_*ih*_ scores do not depend on LD structure using a Wilcoxon test. Histogram of Wilcoxon test statistics comparing the distribution of *Q*_*ih*_ scores for sets of SNPs matched on LD structure of the COPD GWAS SNPs and all other SNPs. The test statistic for the true labeling is in red. The permutation p-value is *P* < 10^−3^ given 10^3^ permutations.
(EPS)

S11 Fig COPD GWAS *Q*_*ih*_ scores do not depend on distance to nearest gene using a KS test. Histogram of KS test statistics comparing the distribution of *Q*_*ih*_ scores for sets of SNPs matched on distance to nearest GSS of the 30 COPD GWAS SNPs and all other SNPs. The test statistic for the true labeling is in red. The permutation p-value is *P* < 10^−3^ given 10^3^ permutations.
(EPS)

S12 Fig COPD GWAS *Q*_*ih*_ scores do not depend on distance to nearest gene using a Wilcoxon test. Histogram of Wilcoxon test statistics comparing the distribution of *Q*_*ih*_ scores for sets of SNPs matched on distance to nearest GSS of the 30 COPD GWAS SNPs and all other SNPs. The test statistic for the true labeling is in red. The permutation p-value is *P* < 10^−3^ given 10^3^ permutations.
(EPS)

S1 Table All network edges for NHGRI-GWAS SNPs. (XLSX)

S2 Table Gene Ontology enrichment results for network communities. (XLSX)

S3 Table All network edges and RegulomeDB scores for COPD-associated SNPs (FDR < 0.05). (PDF)
