# Supplementary material for: Bipartite Community Structure of eQTLs
Source: PLoS Comput Biol. 2016 Sep 12;12(9):e1005033. doi: 10.1371/journal.pcbi.1005033 (PMC5019382; doi:10.1371/journal.pcbi.1005033)
Supplement: S3 Table — (PDF) [file pcbi.1005033.s015.pdf]

RsID	hgnc_symbol	gwas.pval	network.gwas.fdr	regulome.score	SNP Community
rs11012	MAPK8IP1	4.40E-05	0.047394012	1f	39
rs11012	MAPT	4.40E-05	0.047394012	1f	39
rs11012	PLEKHM1	4.40E-05	0.047394012	1f	39
rs11012	LRRC37A2	4.40E-05	0.047394012	1f	39
rs11012	DND1	4.40E-05	0.047394012	1f	39
rs12185268	MAPK8IP1	5.38E-06	0.030303418	1f	39
rs12185268	MAPT	5.38E-06	0.030303418	1f	39
rs12185268	PLEKHM1	5.38E-06	0.030303418	1f	39
rs12185268	LRRC37A2	5.38E-06	0.030303418	1f	39
rs12185268	DND1	5.38E-06	0.030303418	1f	39
rs1378358	KANSL1	4.67E-05	0.047394012	7	39
rs1378358	MAPK8IP1	4.67E-05	0.047394012	7	39
rs1378358	MAPT	4.67E-05	0.047394012	7	39
rs1378358	LRRC37A2	4.67E-05	0.047394012	7	39
rs1378358	DND1	4.67E-05	0.047394012	7	39
rs1396862	KANSL1	3.71E-05	0.045358612	1f	39
rs1396862	MAPK8IP1	3.71E-05	0.045358612	1f	39
rs1396862	MAPT	3.71E-05	0.045358612	1f	39
rs1396862	PLEKHM1	3.71E-05	0.045358612	1f	39
rs1396862	LRRC37A2	3.71E-05	0.045358612	1f	39
rs1396862	DND1	3.71E-05	0.045358612	1f	39
rs142167	MAPK8IP1	3.08E-05	0.045358612	6	39
rs142167	MAPT	3.08E-05	0.045358612	6	39
rs142167	LRRC37A2	3.08E-05	0.045358612	6	39
rs142167	DND1	3.08E-05	0.045358612	6	39
rs1635291	MAPK8IP1	4.30E-05	0.047394012	6	39
rs1635291	MAPT	4.30E-05	0.047394012	6	39
rs1635291	PLEKHM1	4.30E-05	0.047394012	6	39
rs1635291	LRRC37A2	4.30E-05	0.047394012	6	39
rs1635291	DND1	4.30E-05	0.047394012	6	39
rs169201	KANSL1	5.51E-05	0.047394012	6	39
rs169201	MAPK8IP1	5.51E-05	0.047394012	6	39
rs169201	MAPT	5.51E-05	0.047394012	6	39
rs169201	LRRC37A2	5.51E-05	0.047394012	6	39
rs169201	DND1	5.51E-05	0.047394012	6	39
rs16940665	MAPK8IP1	3.07E-05	0.045358612	1f	39
rs16940665	MAPT	3.07E-05	0.045358612	1f	39
rs16940665	PLEKHM1	3.07E-05	0.045358612	1f	39
rs16940665	LRRC37A2	3.07E-05	0.045358612	1f	39
rs16940665	DND1	3.07E-05	0.045358612	1f	39
rs17631676	MAPK8IP1	1.18E-05	0.045358612	6	39
rs17631676	MAPT	1.18E-05	0.045358612	6	39

rs17631676	PLEKHM1	1.18E-05	0.045358612	6	39
rs17631676	LRRC37A2	1.18E-05	0.045358612	6	39
rs17631676	DND1	1.18E-05	0.045358612	6	39
rs17690703	MAPK8IP1	2.70E-05	0.045358612	1b	39
rs17690703	MAPT	2.70E-05	0.045358612	1b	39
rs17690703	PLEKHM1	2.70E-05	0.045358612	1b	39
rs17690703	LRRC37A2	2.70E-05	0.045358612	1b	39
rs17690703	DND1	2.70E-05	0.045358612	1b	39
rs183211	MAPK8IP1	3.03E-05	0.045358612	6	39
rs183211	MAPT	3.03E-05	0.045358612	6	39
rs183211	LRRC37A2	3.03E-05	0.045358612	6	39
rs183211	DND1	3.03E-05	0.045358612	6	39
rs199436	MAPK8IP1	3.37E-05	0.045358612	6	39
rs199436	MAPT	3.37E-05	0.045358612	6	39
rs199436	LRRC37A2	3.37E-05	0.045358612	6	39
rs199436	DND1	3.37E-05	0.045358612	6	39
rs199438	MAPK8IP1	2.93E-05	0.045358612	6	39
rs199438	MAPT	2.93E-05	0.045358612	6	39
rs199438	LRRC37A2	2.93E-05	0.045358612	6	39
rs199438	DND1	2.93E-05	0.045358612	6	39
rs199442	MAPK8IP1	3.34E-05	0.045358612	1f	39
rs199442	MAPT	3.34E-05	0.045358612	1f	39
rs199442	LRRC37A2	3.34E-05	0.045358612	1f	39
rs199442	DND1	3.34E-05	0.045358612	1f	39
rs199444	MAPK8IP1	3.33E-05	0.045358612	7	39
rs199444	MAPT	3.33E-05	0.045358612	7	39
rs199444	LRRC37A2	3.33E-05	0.045358612	7	39
rs199444	DND1	3.33E-05	0.045358612	7	39
rs199448	KANSL1	5.49E-05	0.047394012	6	39
rs199448	MAPK8IP1	5.49E-05	0.047394012	6	39
rs199448	MAPT	5.49E-05	0.047394012	6	39
rs199448	LRRC37A2	5.49E-05	0.047394012	6	39
rs199448	DND1	5.49E-05	0.047394012	6	39
rs199449	MAPK8IP1	3.29E-05	0.045358612	6	39
rs199449	MAPT	3.29E-05	0.045358612	6	39
rs199449	LRRC37A2	3.29E-05	0.045358612	6	39
rs199449	DND1	3.29E-05	0.045358612	6	39
rs199451	KANSL1	5.28E-05	0.047394012	5	39
rs199451	MAPK8IP1	5.28E-05	0.047394012	5	39
rs199451	MAPT	5.28E-05	0.047394012	5	39
rs199451	LRRC37A2	5.28E-05	0.047394012	5	39
rs199451	DND1	5.28E-05	0.047394012	5	39
rs199452	MAPK8IP1	3.03E-05	0.045358612	6	39

rs199452	MAPT	3.03E-05	0.045358612	6	39
rs199452	LRRC37A2	3.03E-05	0.045358612	6	39
rs199452	DND1	3.03E-05	0.045358612	6	39
rs199454	MAPK8IP1	3.53E-05	0.045358612	1f	39
rs199454	MAPT	3.53E-05	0.045358612	1f	39
rs199454	LRRC37A2	3.53E-05	0.045358612	1f	39
rs199454	DND1	3.53E-05	0.045358612	1f	39
rs199456	KANSL1	5.50E-05	0.047394012	1b	39
rs199456	MAPK8IP1	5.50E-05	0.047394012	1b	39
rs199456	MAPT	5.50E-05	0.047394012	1b	39
rs199456	LRRC37A2	5.50E-05	0.047394012	1b	39
rs199456	DND1	5.50E-05	0.047394012	1b	39
rs199457	MAPK8IP1	5.61E-05	0.047394012	5	39
rs199457	MAPT	5.61E-05	0.047394012	5	39
rs199457	LRRC37A2	5.61E-05	0.047394012	5	39
rs199457	DND1	5.61E-05	0.047394012	5	39
rs199501	MAPK8IP1	3.13E-06	0.02644087	1f	39
rs199501	MAPT	3.13E-06	0.02644087	1f	39
rs199501	LRRC37A2	3.13E-06	0.02644087	1f	39
rs199501	DND1	3.13E-06	0.02644087	1f	39
rs199520	MAPK8IP1	1.75E-06	0.02644087	1f	39
rs199520	MAPT	1.75E-06	0.02644087	1f	39
rs199520	LRRC37A2	1.75E-06	0.02644087	1f	39
rs199520	DND1	1.75E-06	0.02644087	1f	39
rs199530	MAPK8IP1	5.97E-06	0.030303418	1f	39
rs199530	MAPT	5.97E-06	0.030303418	1f	39
rs199530	LRRC37A2	5.97E-06	0.030303418	1f	39
rs199530	DND1	5.97E-06	0.030303418	1f	39
rs199533	MAPK8IP1	1.81E-05	0.045358612	1d	39
rs199533	MAPT	1.81E-05	0.045358612	1d	39
rs199533	LRRC37A2	1.81E-05	0.045358612	1d	39
rs199533	DND1	1.81E-05	0.045358612	1d	39
rs199536	MAPK8IP1	5.14E-05	0.047394012	1d	39
rs199536	MAPT	5.14E-05	0.047394012	1d	39
rs199536	LRRC37A2	5.14E-05	0.047394012	1d	39
rs199536	DND1	5.14E-05	0.047394012	1d	39
rs2074404	MAPK8IP1	1.97E-05	0.045358612	1f	40
rs2074404	MAPT	1.97E-05	0.045358612	1f	40
rs2074404	LRRC37A2	1.97E-05	0.045358612	1f	40
rs2074404	DND1	1.97E-05	0.045358612	1f	40
rs2074404	STH	1.97E-05	0.045358612	1f	40
rs7224296	MAPK8IP1	2.44E-06	0.02644087	6	39
rs7224296	MAPT	2.44E-06	0.02644087	6	39

rs7224296	LRRC37A2	2.44E-06	0.02644087	6	39
rs7224296	DND1	2.44E-06	0.02644087	6	39
rs9268528	HLA-DRB5	3.76E-05	0.045358612	1f	33
rs9268528	HLA-DRA	3.76E-05	0.045358612	1f	33
rs9268528	HLA-DRB4	3.76E-05	0.045358612	1f	33

Gene	Community
	39
	39
	39
	39
	39
	39
	39
	39
	39
	39
	39
	39
	39
	39
	39
	39
	39
	39
	39
	39
	39
	39
	39
	39
	39
	39
	39
	39
	39
	39
	39
	39
	39
	39
	39
	39
	39
	39
	39
	39
	39
	39

39
39
39
39
39
39
39
39
39
39
39
39
39
39
39
39
39
39
39
39
39
39
39
39
39
39
39
39
39
39
39
39
39
39
39
39
39
39
39
39
39
39
39

39
39
39
39
39
39
39
39
39
39
39
39
39
39
39
39
39
39
39
39
39
39
39
39
39
39
39
39
39
39
39
39
39
39
39
39
39
39
39
39
40
39
39

39
39
33
33
33
